# Initial Experience of Implementing a Pre-treatment Dry Run for HyperArc Stereotactic Radiosurgery Treatments With Optical Surface Imaging for Intra-fraction Motion Monitoring

**DOI:** 10.7759/cureus.73124

**Published:** 2024-11-06

**Authors:** Eric A Wright, Nathan Becker, Benjamin Mou, Derek Hyde

**Affiliations:** 1 Medical Physics, Hudson Regional Cancer Program, Royal Victoria Regional Health Centre, Barrie, CAN; 2 Medical Physics, BC Cancer Kelowna, Kelowna, CAN; 3 Radiation Oncology, BC Cancer Kelowna, Kelowna, CAN

**Keywords:** general radiation oncology, medical physics, optical surface imaging, photon stereotactic radiosurgery, surface-guided radiotherapy

## Abstract

Linac-based stereotactic radiosurgery (SRS) with planning target volume (PTV) margins <1 mm has become increasingly common in recent years. Optical surface imaging for surface-guided radiation therapy (SGRT) is often used for intra-fraction motion monitoring during these treatments to facilitate the use of a smaller PTV margin by providing real-time quantitative patient positioning information. However, rotating the couch introduces errors to SGRT-reported translations and rotations that can be problematic for SRS treatments with non-coplanar arcs and very small PTV margins. This work presents a novel approach for decreasing the magnitude of these errors by performing a pre-treatment dry run and capturing reference surfaces with the SGRT system at each couch angle included in the treatment plan. Time from cone beam computed tomography (CBCT) to treatment initiation and total treatment session time were reviewed for 30 single-fraction brain SRS cases treated using this technique to determine the effect of including the dry run on treatment session times.

Out of the 30 cases treated between April 2023 and January 2024, 23 treatments required only a single CBCT prior to treatment, with no additional mid-treatment imaging required to verify patient positioning after motion. The median time between CBCT and treatment initiation was 7.98 minutes (interquartile range (IQR) = 7.28 to 8.93 minutes). The median time from CBCT to treatment completion was 15.43 minutes (IQR = 13.67 to 21.97 minutes). In the six patients that required one additional CBCT, the treatment session times ranged from 24.32 to 32.83 minutes. There was one patient who required three mid-treatment CBCTs, and the treatment session time was 67.87 minutes.

Incorporating the pre-treatment dry run with the acquisition of reference surfaces at each treatment angle decreased errors in SGRT-reported translations and rotations associated with couch rotation without significantly increasing treatment session times.

## Introduction

Linac-based stereotactic radiosurgery (SRS) has been adapted into routine workflows for the high-precision treatment of brain metastases, acoustic neuromas, arteriovenous malformations, trigeminal neuralgia, and other intracranial targets [1-4]. Stereotactic radiosurgery relies on advanced imaging for accurate target localization to reduce the planning target volume (PTV) margins, thereby reducing the volume of irradiated normal brain tissue. Multiple studies demonstrate that PTV margins larger than 1 mm do not improve local recurrence rates but can increase the risk of symptomatic radiation necrosis [5,6].

In recent years, surface-guided radiation therapy (SGRT) systems have gained prominence as a non-invasive solution for intra-fraction motion monitoring. Surface-guided radiation therapy systems rely on three ceiling-mounted camera-projector pods to provide quantitative, real-time patient positioning information using stereoscopic imaging of a speckled light pattern projected onto the patient. The system matches the recorded patient surface to a reference position and reports vertical, longitudinal, and lateral offsets between the current position and the reference, as well as the vector sum of all three translations (i.e., magnitude). This facilitates the use of smaller PTV margins for stereotactic radiotherapy [7] by allowing treatment to be paused if the patient’s surface deviates from the treatment position by a magnitude greater than a predetermined threshold. Surface-guided radiation therapy used during SRS allows PTV margins to be reduced to as small as 1 mm while using far less invasive immobilization than traditional frame-based methods [8].

One drawback of using SGRT for linac-based SRS is errors introduced at non-coplanar couch angles [9]. Experiments using an anthropomorphic head phantom indicate translational errors of up to 0.5-0.6 mm at couch angles of 45°-90° [10]. Errors of this magnitude in SGRT-reported translations are problematic when monitoring for motion less than 1 mm in SRS applications since they can result in unnecessary breaks in treatment delivery. This technical report describes our institutional experience with a novel approach to decrease the magnitude of SGRT errors at non-zero couch angles during SRS treatments with non-coplanar arcs.

## Technical report

HyperArc planning

**Figure 1:**
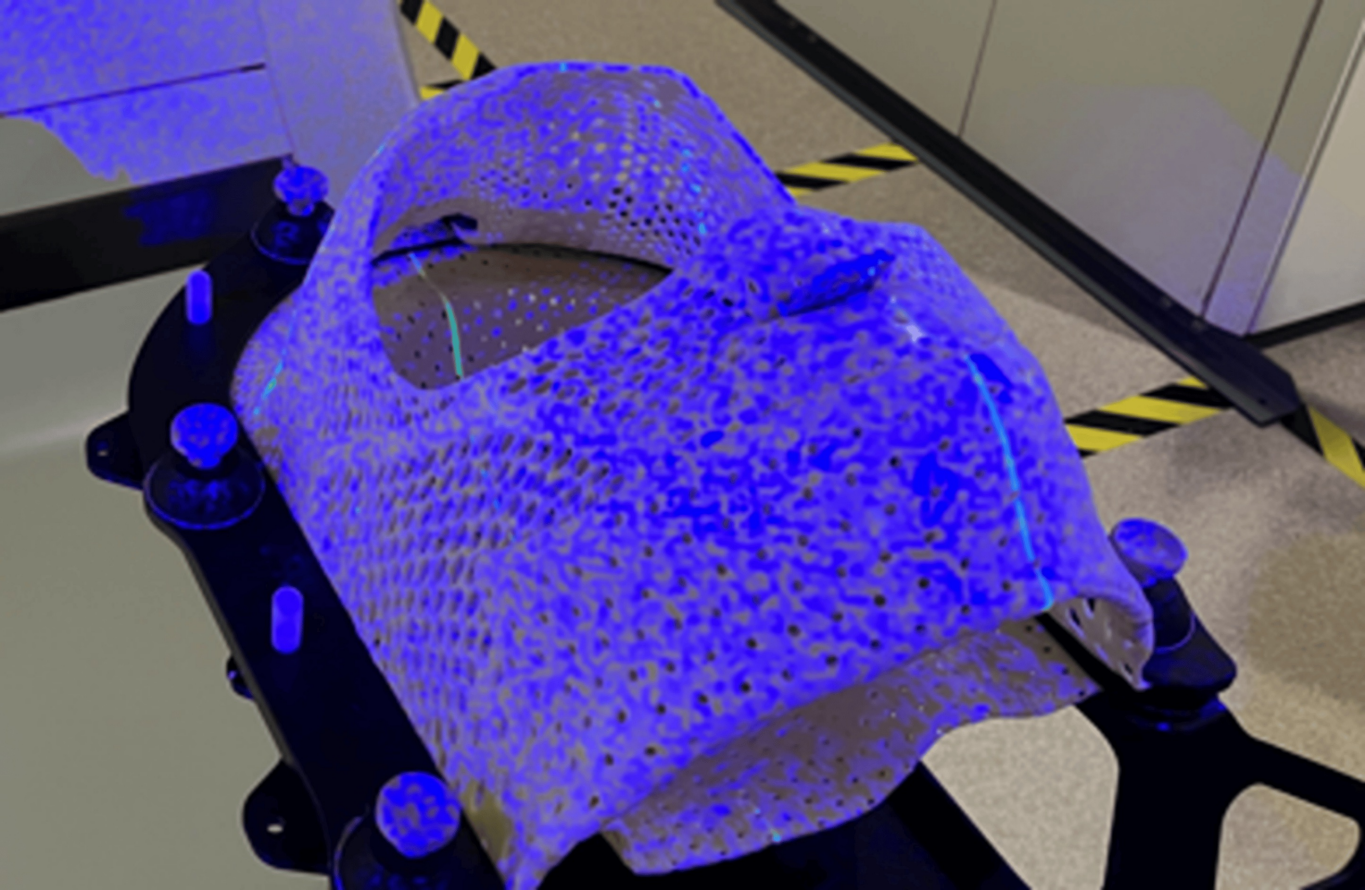
Encompass open-faced thermoplastic shell An example of the open-faced thermoplastic shell used with the Encompass system to facilitate optical surface imaging with Identify using stereoscopic imaging of the blue speckled light pattern. The open region used for motion monitoring goes from just above the eyebrows to just below the nose. There is also a biteblock incorporated into the region of the mask covering the mouth to improve inter- and intra-fraction positioning reproducibility.

For computed tomography (CT) simulation, patients were immobilized on the Encompass SRS Immobilization System (CQ Medical, Avondale, PA), which uses an open-face mask to facilitate patient setup and intra-fraction monitoring with an SGRT system (Figure 1). The planning CT was imported into Aria v15.6 (Varian Medical Systems, Palo Alto, CA) and fused with a magnetic resonance image (MRI). Gross tumor volumes (GTVs) were contoured using a high-resolution gadolinium-enhanced T1-weighted MRI, and a 1 mm isotropic PTV margin was applied to the GTV. Plans were generated using Hyperarc (Varian Medical Systems), an automated tool for planning SRS treatments with non-coplanar arcs. All plans used a single isocentre and consisted of one full or partial arc at a couch angle of 0°, plus two partial arcs at couch angles of 45° and 315°. Prescription doses ranged from 14 Gy [11] to 22 Gy [12] in 1 fraction. Plans were optimized to cover 99% of the GTV with 100% of the prescription dose and 99% of the PTV with 90% of the prescription dose.

Pre-treatment identify workflow

Patients were treated on a TrueBeam linac equipped with a PerfectPitch 6-degree of freedom (DoF) couch and Identify (v2.2) SGRT system (Varian Medical Systems). DICOM plan and structure sets were exported from Eclipse to Identify. The region of interest (ROI) was contoured in Eclipse to include the entire portion of the face visible through the opening in the facemask. Prior to treatment, this ROI was copied onto the external contour from CT simulation using the Planning Tool on the Identify Workstation, and the monitoring thresholds were adjusted to 1 mm and 1^o^.

Patients were set up to match the region of the face covered by the monitoring ROI to its corresponding position on the external contour from CT simulation within 1 mm and 1^o^. Cone-beam CT (CBCT) was performed, and imaging arms were retracted to the mid position at scan conclusion to avoid obscuring the Identify camera pods on either side of the gantry. Automated 6-DoF registration was performed to match the skull to the planning CT. If rotations exceeded 2^o^, then another CBCT was acquired after applying shifts to verify patient positioning.

After applying shifts, a reference surface was saved on the Identify handheld with the couch in the treatment position it will be in during delivery of the first arc (planned for 0^o^ couch angle). The couch was then rotated from the control area to the angle required for the second arc (e.g., 315^o^ plus the rotation applied during the image match). A reference surface was saved in the treatment position for delivery of the second arc. This process was repeated for each non-coplanar arc until reference surfaces were saved in the treatment position for all arcs in the plan.

**Figure 2:**
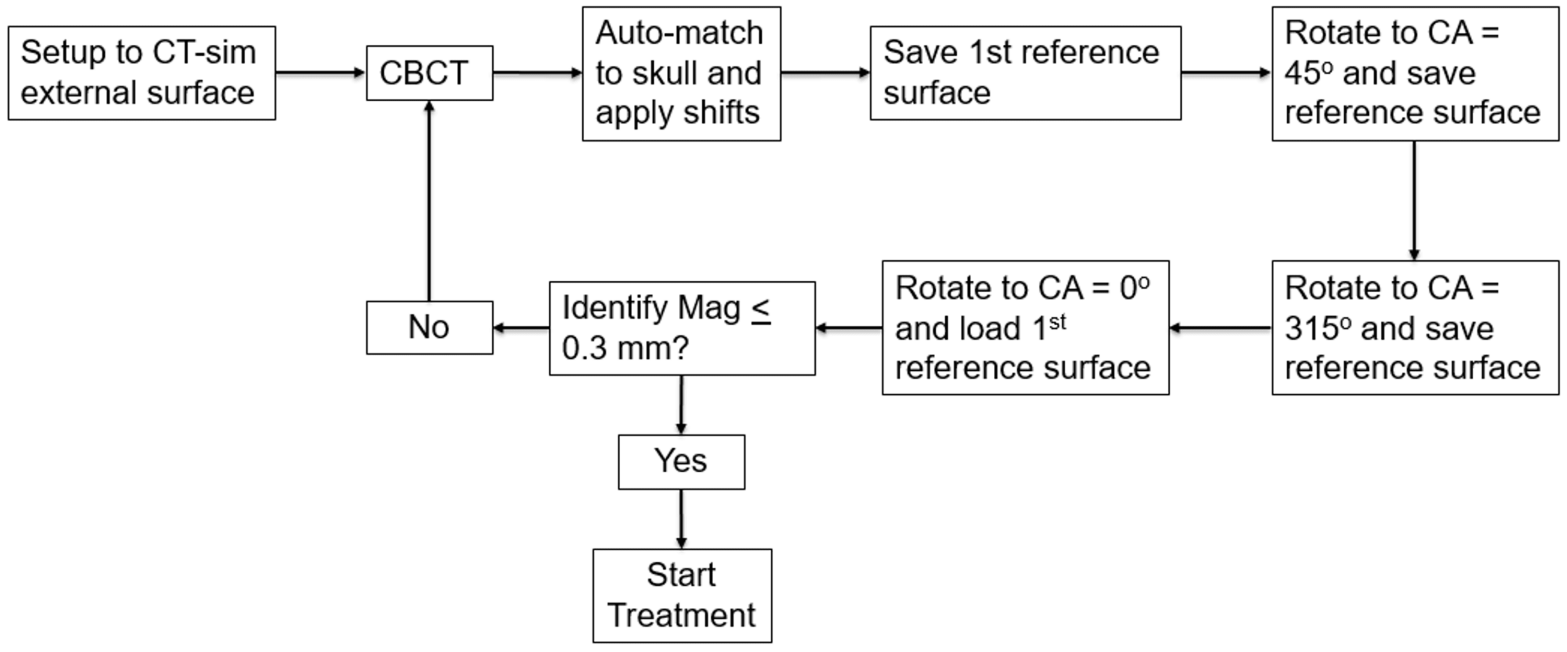
Pre-treatment dry run process flowchart Summary of the workflow for patient setup, cone-beam CT (CBCT) acquisition, image match, and pre-treatment dry run where Identify reference surfaces were acquired at each couch angle (CA) in the treatment plan.

After saving reference surfaces for all non-coplanar arcs, the couch was returned to the initial position, and the reference surface acquired immediately after the CBCT match was loaded using the Identify handheld to verify patient positioning prior to starting treatment. If the vector sum of all translations (i.e., the Mag_cm_ parameter on Identify) was <0.3 mm, then treatment delivery was initiated. If Identify Mag_cm_ was > 0.3 mm, then CBCT was reacquired, and the process was repeated. This process is summarized by the flowchart in Figure 2.

Treatment with identify intra-fraction motion monitoring

HyperArc allows for automated treatment delivery. After each arc is finished, the couch automatically rotates into the position required for the next arc. The handheld was used to switch between the reference surfaces corresponding to each arc while the couch was rotating into position. Treatment was paused if Identify-reported translations or rotations exceeded 1mm/1o for greater than 2-3 seconds. If the translations/rotations dropped below the 1mm/1^o^ threshold within a reasonable timeframe, then treatment was continued. If the change in surface position persisted beyond 20-30 seconds, then the gantry was rotated to 0° or 180° to ensure the Identify cameras on either side of the couch were not blocked by the gantry. If moving the gantry out of the camera field of view (FOV) brought translations/rotations back below 1mm/1°, then treatment was allowed to continue provided the Identify Magcm parameter did not increase further. If moving the gantry out of the camera FOV did not resolve the difference in surface position, then the couch was returned to 0° and a mid-treatment CBCT was acquired to verify patient positioning. Surfaces were re-acquired if shifts were applied.

Results

**Figure 3:**
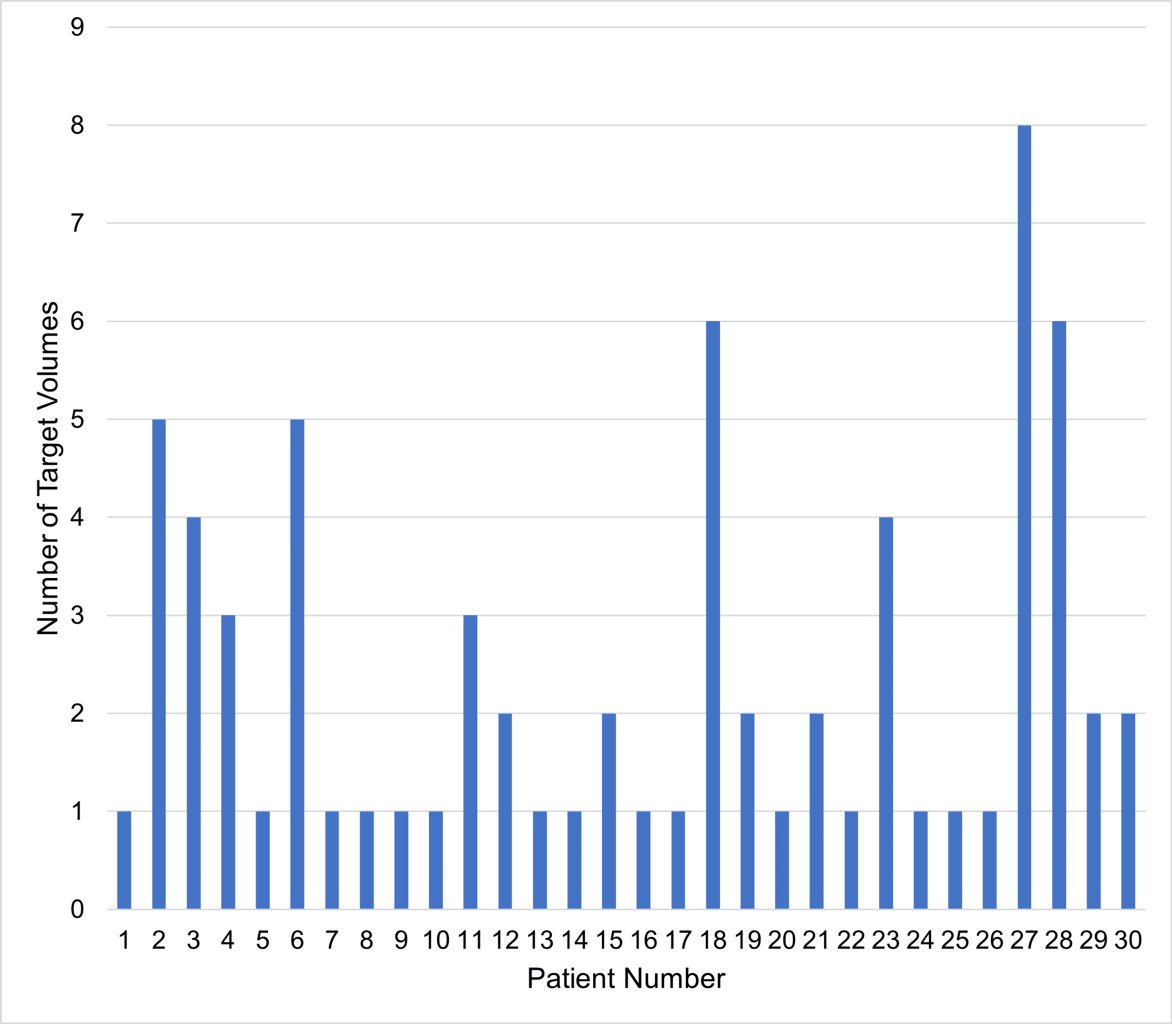
Target volumes treated per patient The number of target volumes per treatment ranged from one to eight. The average number of target volumes was 2.4 and the standard deviation was 1.9.

Thirty patients were treated with SRS between April 2023 and January 2024. The number of target volumes treated ranged from one to eight, with an average of 2.4 and a standard deviation of 1.9 (Figure 3). Twenty-three patients (77%) only required a single CBCT prior to treatment. One patient had an additional CBCT acquired before starting treatment, five patients had one additional mid-treatment CBCT, one patient had one additional CBCT before starting treatment, and two mid-treatment CBCTs.

The median time required for CBCT, image matching, setup adjustment, and acquisition of reference surfaces at each couch angle was 7.98 minutes (interquartile range (IQR) = 7.28 to 8.93 minutes). This time was increased to 22.06 and 24.32 minutes in the two patients who required an additional pre-treatment CBCT prior to treatment initiation.

**Figure 4:**
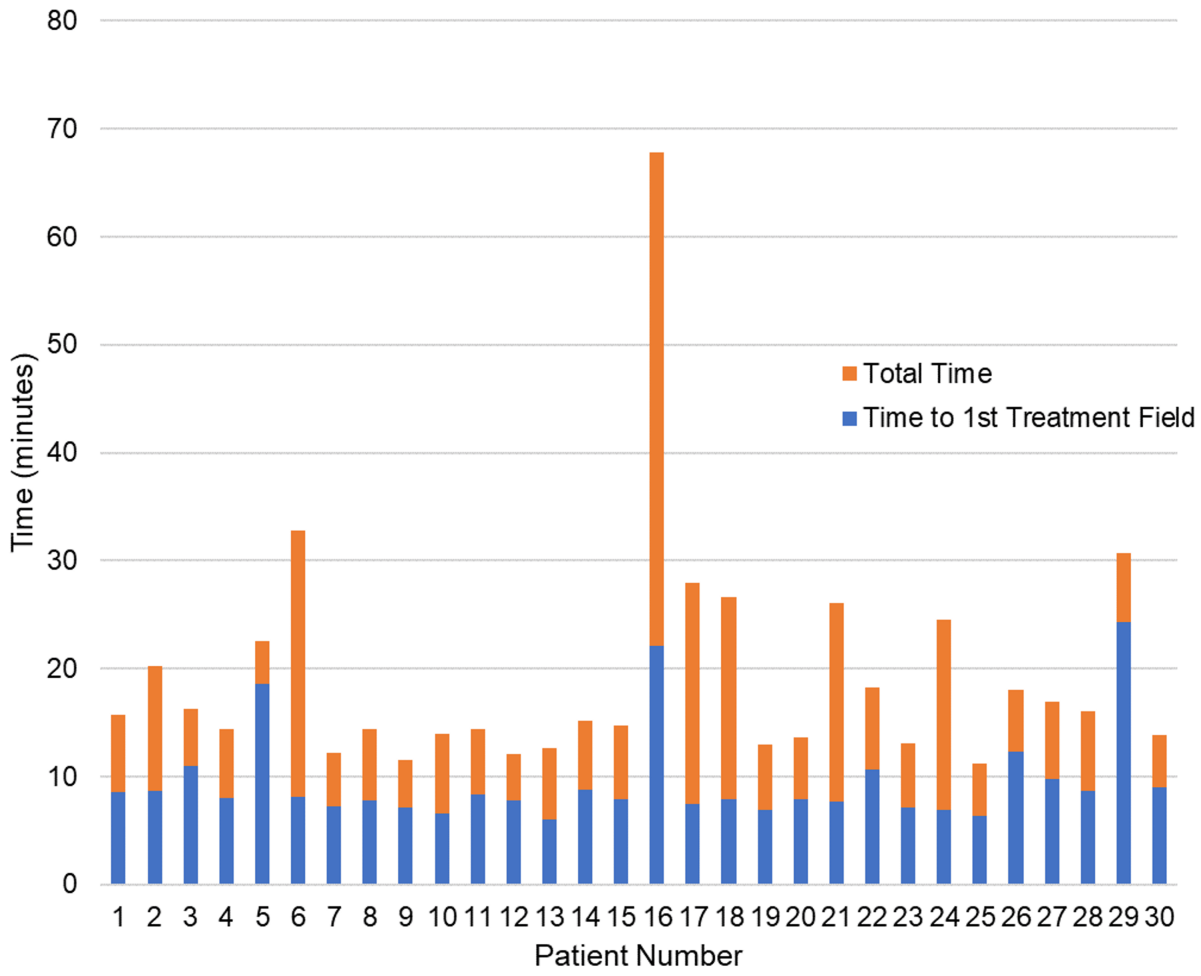
Treatment session times The length of time between starting cone-beam CT (CBCT) and starting treatment for each patient is represented by the blue bar. This time covered CBCT acquisition, image matching, adjustment of patient positioning, and saving Identify reference surfaces at each couch angle included in the treatment plan. The length of time from the start of the first treatment beam to the end of the treatment is represented by the orange bars.

The total time from CBCT to finishing treatment had a median value of 15.43 minutes (IQR = 13.67 to 21.97 minutes). In the six patients that required only one additional CBCT (either before or after initiating treatment), total CBCT to end treatment time ranged from 24.32 to 32.83 minutes. In the one patient that required one additional pre-treatment CBCT and two mid-treatment CBCTs, the total CBCT to end treatment time was 67.87 minutes. Times from the start of CBCT to initiating treatment and times from initiation to end of treatment delivery for each patient are summarized in Figure 4. Delays in patients that required additional mid-treatment CBCTs came from the additional time needed to rotate the couch back to 0o (if motion occurred during the second or third partial arc), acquire and reconstruct the mid-treatment CBCT, perform image match and apply shifts, and re-acquire the reference surfaces for Identify at the new patient position.

## Discussion

Linac-based SRS treating multiple targets with a single isocentre is becoming increasingly common [13-15]. Reducing PTV margins is critical for these treatments to reduce the volume of normal brain irradiation to decrease the risk of symptomatic radiation necrosis. Studies have shown that the volume of a normal brain receiving 5, 8, 10, and 12 Gy approximately doubles when the PTV margin is increased from 0 to 1 mm and triples when the PTV margin increases from 0 mm to 2 mm [16]. This has led some institutions to plan with no PTV margin [4]. Early results have found similar local control rates [17]. Increasing the precision and accuracy of SGRT monitoring for intra-fraction motion will take on increased importance for these treatments with 0 mm to 1 mm PTV margins.

This report summarizes a novel workflow developed at our clinic to reduce the error in SGRT-reported translations at non-zero couch angles by incorporating a pre-treatment dry run. These preliminary results indicate that treatment can still be completed within a reasonable timeframe in most patients. There was one outlier whose treatment session took 67.9 minutes. The Encompass immobilization system includes a bite block in the portion of the thermoplastic mask over the mouth, which is included to make it easier for patients to remain motionless for the duration of treatment. The bite block could not be used for this patient because they did not have teeth or dentures, and as a result, the patient had difficulty maintaining a consistent position. Identify detected changes in positioning that were outside tolerance values once before initiating treatment and two additional times after initiating treatment, and additional CBCTs were acquired each time according to the protocol summarized in Figure 2. The additional time required to acquire CBCTs, perform image matching, apply shifts, and re-acquire reference surfaces for Identify for each additional CBCT resulted in a much longer treatment session relative to the other 29 patients included in the manuscript.

The American Association of Physicists in Medicine (AAPM) Task Group (TG) 302 report on SGRT [18] recommends acquiring a new SGRT reference surface immediately after performing a CBCT match and applying shifts. This single reference surface is then used for intra-fraction motion monitoring for the entire treatment, including arcs delivered at non-zero couch angles by virtually rotating the reference surface about the isocentre in Identify to match the couch angle in the plan. Theoretically, virtually rotating the reference surface to match the couch angle in the plan should result in Identify measuring only small translations caused by couch walkout, assuming there is no change in patient positioning. However, SGRT-reported translations at couch angles of ±45° and 90° are also affected by discrepancies in the position reported by each of the three camera-projector pods.

Surface-guided radiation therapy systems are comprised of three ceiling-mounted camera-projector pods, one located at the foot of the couch and one located on the right and left sides of the couch. Each camera-projector pod is individually capable of stereoscopic localization, but it is necessary to have three pods placed at different locations in the bunker so that a large enough area on the patient's surface is always visible. However, the three camera-projector pods do not agree perfectly. There will always be sub-millimeter discrepancies in the position of a phantom reported by one camera-projector pod versus another. Rotating the couch away from the angle where the reference surface was initially acquired changes what parts of the monitoring ROI are visible to each of the three camera-projector pods, and since the three camera-projector pods do not agree perfectly, this manifests as the SGRT system incorrectly reporting sub-millimeter translations that have not truly occurred. There is a similar effect when the gantry head blocks one of the SGRT cameras, since this suddenly cuts off information from one of the camera-projector pods, forcing the system to rely entirely on the other two, which will not perfectly agree with the one that has been blocked by the gantry head.

Previous studies using a similar setup with Identify and a Varian c-arm linac have shown translational errors of up to 0.5 mm to 0.6 mm at couch angles of 45° to 90° [9,10]. These studies used an anthropomorphic head phantom with an implanted metal ball bearing (BB), which was set at couch angle 0o, with the BB at isocentre. Initial phantom surface and BB position were captured using Identify and portal imaging. The couch was rotated to different angles while Identify monitored, and a portal image was acquired at each couch angle. The difference between translations reported by Identify at each couch angle and couch walkout determined by measuring the change in (BB) position on portal images was reported as an error in SGRT-reported translation values. These experiments were repeated at our center, and similar results were observed.

This is a well-known issue with SGRT, and it is acknowledged in AAPM TG-302. The recommendation is for a qualified medical physicist to determine whether SGRT-reported offsets are caused by real patient motion, couch rotation effects, or gantry blockage effects. The proposed solution is to pause treatment every time the SGRT-reported translation magnitude exceeds 1 mm, then rotate the gantry head out of the SGRT camera field-of-view to rule out gantry blockage. If the magnitude still exceeds 1 mm, then rotate the couch back to 0^o^ to rule out couch rotation effects. If the magnitude still exceeds 1 mm at couch angle 0o, then mid-treatment CBCT should be performed to verify positioning. Conversely, if the magnitude drops below 1 mm, then it is reasonable to assume that the SGRT-reported translations were caused by a combination of couch rotation effects and gantry blockage effects and that it is safe to proceed with treatment provided that the SGRT-reported offsets do not increase further in magnitude [18].

Our standard institutional practice is to set the threshold for SGRT intra-fraction motion monitoring equal to the PTV margin. When the magnitude of error in SGRT-reported translations approaches the PTV margin, it is problematic for two reasons. Firstly, if there is a real change in patient positioning but in the opposite direction from the translations caused by couch rotation effects and/or gantry blockage effects, the magnitude of the position change will be greater than what is reported by the SGRT system. For example, consider a scenario where rotating the couch to 45° causes Identify to incorrectly report a 0.6mm shift to the right due to couch rotation effects, even though there has been no real change in patient positioning. If the patient were to start moving to the left, they would be able to move by up to 1.6 mm before the threshold for pausing treatment was reached (i.e., first 6 mm to cancel out the error caused by couch rotation, then another 1 mm to reach the 1 mm threshold). Secondly, real changes in patient positioning in the same direction as the errors caused by couch rotation effects and/or gantry blockage effects will result in unnecessary beam interruptions when the patient positioning has changed by <1 mm. In the same scenario as what is outlined above, if the patient were to start moving to the right instead of the left, the 1 mm threshold would be reached after only a 0.3 mm change in positioning. This leads to beam delivery being paused to return to couch angle 0°, only to find that the patient positioning has barely changed. These unnecessary interruptions may occur frequently when the level of error in SGRT-reported translations approaches the 1 mm monitoring threshold for pausing treatment. The root cause of these problems is the error in translational offsets that is introduced when the couch is rotated away from the angle where the reference surface was acquired. The novel workflow addresses this by acquiring separate reference surfaces for each couch angle used in the plan, each acquired at the couch angle where beam delivery will take place, effectively eliminating this source of error.

The limitations of this method are the risk of the patient moving before all reference surfaces are acquired. This risk is mitigated by verifying patient positioning after the dry run using the first reference surface acquired. Therefore, it is critically important that this first reference surface is acquired promptly after completing shifts to minimize the chance of any motion occurring in between the application of shifts and the acquisition of the first reference surface. Another drawback to this method is that any actual translations that occur when the couch is rotated due to couch walkout are also zeroed out when the new reference surfaces are captured. Couch rotation walkout is generally small compared to the SGRT errors caused by rotating the couch and can be tracked and accounted for with routine quality assurance. The last limitation is the additional time required for the dry run and the acquisition of reference surfaces at each couch angle. The results of this paper indicate that most treatment sessions are still completed within a reasonable timeframe.

## Conclusions

A novel approach to decreasing the magnitude of SGRT errors at non-zero couch angles during non-coplanar linac-based SRS can be achieved by incorporating a pre-treatment dry run and acquiring reference surfaces at each couch angle used in the treatment plan. Treatment sessions were completed within a reasonable timeframe. Improvements in SRS precision can potentially lead to further PTV margin reductions and lower toxicity rates without compromising disease control.
